# Retrospective Illumination Correction of Retinal Images

**DOI:** 10.1155/2010/780262

**Published:** 2010-07-04

**Authors:** Libor Kubecka, Jiri Jan, Radim Kolar

**Affiliations:** The Faculty of Electrical Engineering and Communication, Brno University of Technology, 60200 Brno, Czech Republic

## Abstract

A method for correction of nonhomogenous illumination based on optimization of parameters of B-spline shading model with respect to Shannon's entropy is presented. The evaluation of Shannon's entropy is based on Parzen windowing method (Mangin, 2000) with the spline-based shading model. This allows us to express the derivatives of the entropy criterion analytically, which enables efficient use of gradient-based optimization algorithms. Seven different gradient- and nongradient-based optimization algorithms were initially tested on a set of 40 simulated retinal images, generated by a model of the respective image acquisition system. Among the tested optimizers, the gradient-based optimizer with varying step has shown to have the fastest convergence while providing the best precision. The final algorithm proved to be able of suppressing approximately 70% of the artificially introduced non-homogenous illumination. To assess the practical utility of the method, it was qualitatively tested on a set of 336 real retinal images; it proved the ability of eliminating the illumination inhomogeneity substantially in most of cases. The application field of this method is especially in preprocessing of retinal images, as preparation for reliable segmentation or registration.

## 1. Introduction

Improper scene illumination as well as nonideal acquisition conditions due to for example, misadjusted imaging system can introduce severe distortions into the resulting image. These distortions are usually perceived as smooth intensity variations across the image. According to the terminology commonly used in processing of magnetic resonance (MR) images, we call these systematic intensity level inhomogeneities as the *bias field*. With such unevenness, the subsequent image processing like image registration, segmentation, or pattern recognition may be substantially complicated; therefore, the correction of illumination inhomogeneities is highly desirable. Unfortunately, separating the bias field from the true underlying image is an under-constrained and ill-posed problem with fewer observations than free variables. For this reason, regularization of the problem is necessary. 

 Most existing bias correction methods assume that the bias field is multiplicative, slowly varying, and tissue independent. Many techniques ignore the noise and apply a log transform to make the bias field additive. The known illumination correction methods can be categorized in the following groups: filtering, segmentation based, surface fitting, and other methods. 

Illumination inhomogeneities are generated during acquisition process in systems with different modalities. Here, the proposed illumination correction method will be applied on retinal images from confocal scanning laser ophthalmoscope (CSLO). This correction is an important pre-processing task in image segmentation and/or multimodal registration [1–4]. 

The earliest bias correction techniques were based on phantoms [5] with known structure; this approach enables estimation of the bias field, its inversion and removal. Provided the calibrated phantom is available, this method can be considered the ground truth but in real situations is not often applicable.

Linear filtering methods [6, 7] try to estimate and eliminate the additive bias of the image using unsharp masking with defocus larger than any object in the image. These techniques can be extended, via nonlinear morphological approach to the multiplicative bias field estimation [6]. Homomorphic unsharp filtering methods [8–10] assume a separation of the low frequency bias field from the higher frequencies of the image structures. 

Some simple methods, as [11], require expert supervision, which is time-consuming and often too subjective. In this approach, the expert specifies some parameters related to the intensity models of the different tissues and forms a list of intensity values and locations related to the background or to object classes. Then, the bias field over the image may be obtained by least-squares fitting to the intensity values at the preselected points. These methods are closely connected to segmentation-based methods. Authors of [12] combine shading correction with adaptive segmentation. They use a fuzzy C-means algorithm to allow labeling of a pixel to be influenced by its immediate neighbors. Main benefits of this approach are high robustness to salt-and-pepper noise and computational efficiency of the algorithm.

The expectation maximization (EM) algorithm is proposed in [13] to compute iteratively the optimal smooth bias field corrupting the data based on classification into several tissue classes. Their formulation includes the bias distortion in the statistical model of the pixel distribution, that is, the bias field influences the distribution by locally changing its mean value. The algorithm iterates two steps, the E-step calculating the posterior tissue probabilities, and the M-step estimating the bias field. The Styner's method [14] is based on a simplified model of the imaging process, a parametric model of a tissue class statistics, and a polynomial model of the inhomogeneity field. The estimation of the parametric bias field is formulated as a non-linear energy minimization problem solved by an evolution strategy. 

Segmentation-based approaches raise the problem of selecting the number of classes, which have to be explicitly modeled. Furthermore, these algorithms unfortunately tend to converge to a local nonoptimal minimum for some bias configurations, especially when more than two tissue classes are modeled [15]. The main problem of segmentation-based technique is that the determination of specific class-conditioned intensity is difficult when the image is incorrectly illuminated; however, the illumination correction is the main aim. Also, these methods usually assume that the intensity distribution of an image is normal and given by distributions of individual tissues, which may be often invalid. The above drawbacks are even more serious when correcting pathological image data. Therefore, Likar et al. [16] used the closed connection between intensity nonuniformity correction and segmentation, and proposed a method, which iteratively interleaves them, so that both of them gradually improve, until the final correction and segmentation is reached. The method is based on iterative minimization of the class square-error of intensity distributions caused by non-uniformity and on a nonparametric segmentation method. The method makes no assumption on the distribution of tissue intensity and does not require initialization. 

To overcome the segmentation problems, bias correction methods not requiring the segmentation were designed based on a chosen image quality criterion. In [17], a presumed histogram matching method is presented. Authors of [18] suggested the iterative optimization method, which seeks the smooth multiplicative field that maximizes the higher frequency content of the distribution of tissue intensity. It requires a parametric model for the bias field but not a decomposition of the intensities. The quality criterion derived from the information theory has been also used in several applications. In [19], a polynomial multiplicative and additive shading model was introduced employing the cost-function based on image entropy and Powell optimizer. Mangin [20] uses image entropy combined with a measure of the field smoothness as the image quality cost function. Authors of [14] model the bias field using Legendre polynomials and use the energy function based on a multiclass model estimator and evolutional search algorithm.

Specific methods of illumination correction were proposed in the frame of retinal image processing and analysis. Simple and fast methods using large-kernel median filter to obtain a low-pass correction coefficients were used for CSLO image preprocessing in [21]. Authors of [22] model the bias field (background image) of a fundus (basic retinal) image as a white Gaussian random field and use Mahalanobis distance for background pixel classification. Contrast normalization using high-pass filtered image is used in [2] as one step of microaneurysm detection procedure. Additive model of nonuniform illumination is used in [3], together with adaptive histogram equalization. 

Other approaches exist, for example, in applications including illumination correction for face recognition [23, 24] and restoration of digitized documents [25, 26]. The latter application frequently uses multiplicative model and specific properties of the processed images (e.g., illumination edges or geometrical distortion). 

In this paper, we focus our attention on methods estimating the parametric illumination field using the quality criterion derived from the information theory. We use a multiplicative model of nonuniform illumination and parametric local bias model for formulation of criterion function and its derivatives (Section 2). The results are presented in Section 3 for different optimizers, which were tested for two image sets—artificially illuminated images and real CSLO images. The assessment of the results concludes the paper in Section 4.

## 2. Methods

### 2.1. Acquisition Model

**Figure 1:**
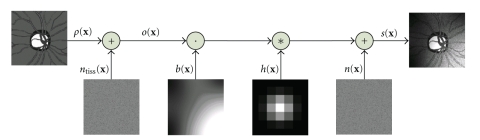
Model of image acquisition. Ideal image is corrupted by noise, multiplicative illumination, and finite resolution of the imaging device (characterized by Gaussian point spread function).

For the purpose of illumination correction process, we need a model of image creation. We assume, that each tissue class (vessels, optic disc, retinal surface) has a different mean value *ρ*(**x**) of the property measured by the imaging device. Moreover, every class of tissue has a characteristic texture, which can be modeled by the additive noise *n*_tiss_(**x**). The ideal output signal *o*(**x**) therefore consists of piecewise constant values plus additive noise. Due to finite size of the point spread function *h*(**x**) of the imaging device (different from the ideal Dirac impulse), this ideal signal is corrupted by convolution with *h*(**x**) and with the noise generated by the device *n*(**x**) (thermal or electronic noise and the noise coming from digitization during acquisition process). The overall equation of the observed image data can be formalized as follows (*b*(**x**) being the illumination), see Figure 1:

(1) $$\begin{array}{cc} s\left(x\right) & =\left[\left(\rho \left(x\right)+{n_{\text{tiss}}}_{}\left(x\right)\right)b\left(x\right)\right]{}^{*}h\left(x\right)\\ & \;+n\left(x\right)\approx o\left(x\right)b\left(x\right)+n\left(x\right). \end{array}$$

On the right-hand side of (1), we neglected the smoothing due to the imaging properties not playing a substantial role in our problem (the disturbance need not be taken into account in the retinal applications).

**Figure 2:**
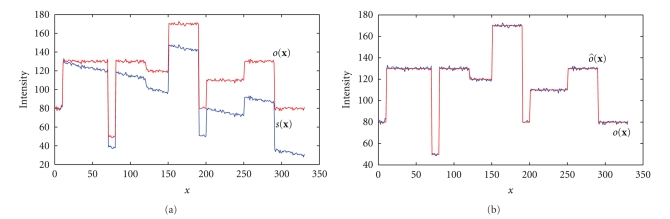
(a): Example of a line of input signal: *o*(**x**) is the ideal signal with the texture noise, *s*(**x**) is acquired signal furthermore corrupted by non-homogenous illumination (b): results of the correction process, *o*(**x**)—ideal signal, $\hat{o}(x)$— signal after entropy-based correction (nearly ideally recovered).

Thus, under the assumption that the bias involved in image creation process is multiplicative, and that it is the only important disturbance, we can reconstruct the original signal as

(2) $$\hat{o}\left(x\right)=\frac{s\left(x\right)}{\hat{b}\left(x\;|\;\Phi \right)}-\frac{n\left(x\right)}{\hat{b}\left(x\;|\;\Phi \right)}\approx \frac{s\left(x\right)}{\hat{b}\left(x\;|\;\Phi \right)}=s\left(x\right){\hat{b}}^{-1}\left(x\;|\;\Phi \right),$$

where $\hat{b}\left(x\right)$ is the optimal illumination bias model controlled by parameters Φ_*i*_ forming the vector Φ, while ${\hat{b}}^{-1}\left(x\right)$ is its reciprocal value. Example of a line input signal (measured line profile) can be seen on Figure 2. The choice of a proper bias model *b*(**x**) as well as of an appropriate criterion function, evaluating how well the non-homogenous illumination of the image is corrected for current values of the bias model parameters, is crucial.

### 2.2. Bias Model

In this section, we extend the Likar's algorithm [19] and applied the modified algorithm for retrospective shading correction of retinal images. We derive an expression for the criterion of quality of illumination compensation based on Shannon's entropy and on Parzen windowing probability estimation. Further, as a novelty, we derive analytical expressions for derivatives of this criterion with respect to parameters of the used B-spline multiplicative illumination model. The analytical expressions ease substantially the optimization calculations compared to purely numerical evaluation of derivatives.

Generally, in accordance with [14, 19], the intensity transformation performed by the reciprocal bias model can be defined by a linear combination of *K* smooth basis functions *r*_*i*_(**x**).

(3) $${\hat{b}}^{-1}\left(x\right)=\overset{K}{\underset{i=1}{\sum }}\Phi_{i}r_{i}\left(x\right),$$

where Φ_*i*_ are the parameters of the transform defining the contribution of each basis. 

In order to regularize the problem of finding the bias field that would optimally correct the illumination inhomogeneity, we used the bases in the following form:

(4) $$r_{i}\left(x\right)=\frac{q_{i}\left(x\right){-}^{m}c\left(x\right)}{{}^{n}c\left(x\right)},$$

where *q*_*i*_(**x**) are smooth polynomial basis functions, ^*m*^ *c*(**x**) is a mean correction coefficient, and ^*n*^ *c*(**x**) is a normalization coefficient. The correction coefficients are defined using constraints presented in [19], where the mean preserving condition preventing a global shift of the mean intensity of the resultant corrected image is formulated as

(5) $$\frac{1}{\Theta }\underset{x\in \Omega }{\sum }s\left(x\right)=\frac{1}{\Theta }\underset{x\in \Omega }{\sum }s\left(x\right){\hat{b}}^{-1}\left(x\right).$$

Here, Θ is the size of the image domain Ω (or region of interest: ROI) in pixels. Further, as we use the multiplicative bias model, the final intensity is differently sensitive to values of different parameters, thus producing non-linear logarithmic scale (see [19] for detailed description). This fact is undesirable in optimization. Therefore, to assure that every parameter has the same influence on the intensity transform, a normalization constraint for every base function kernel *r_i_*(**x**) is introduced as

(6) $$\frac{1}{\Theta }\underset{x\in \Omega }{\sum }\left|s\left(x\right)r_{i}\left(x\right)\right|=1,\;\forall i,$$

We have found that the illumination distortion of retinal images shows a significant spatial variance, while both illumination models mentioned in [14, 19] have rather a global character influencing the whole image despite possibly only locally defined distortions (e.g., when the image is corrupted in its upper left corner only, the polynomial model may significantly influence the opposite corner as well). Therefore, to model such local distortions, high-order models have to be used, which results in a high number of parameters to optimize, besides a danger of oscillatory character of the functions.

Hence, we decided to use the locally defined mean-corrected and normalized reciprocal bias model based on B-splines, formalized as follows:

(7)   b^−1(x)=1+∑xi∈PΦ(xi)[(β(d)(Δxi)−c(xi)  m)  nc(xi)],       where  Δxi=x−xih.

**Figure 3:**
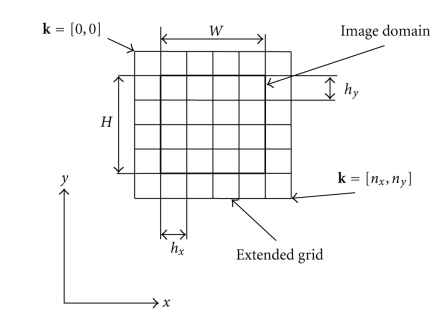
Formation of grid of control points for B-spline intensity transform.

For the case of two dimensions, *β* ^(*d*)^(**x**) = *β* ^(*d*)^(*x*_1_) · *β* ^(*d*)^(*x*_2_) is a separable *d*th order B-spline kernel, *P* is a set of *n_x1_* × *n_x2_* control points of the transform regularly deployed over the extended image domain Ω with the spacing **h=[** *h_1_*, *h_2_* **]** (*h_k_* possibly different but constant for each dimension, see Figure 3 for illustrative description). The division indicated in the expression for Δ**x** in (7) should be understood element-wise; thus **x** *_i_*/**h** are the integer control-point indices. The Φ(**x** *i*) = *ϕ*_*i*_ are scalar coefficients corresponding to the control point. These coefficients are the parameters of the bias model and express the weight of influence of each B-spline basis function. As we use the 3rd order B-spline, which is nonzero only for Δ*x*_*k*_ < −2,2>*∈*, only 4 × 4 basis functions influence the computation of a current point **x** for two dimensions. For efficient implementation, the grid of control points has to be extended beyond the image borders. ^*m*^ *c*(**x** *i*) = ^*m*^ *c*_*i*_ are coefficients ensuring the mean conservation condition (5) and ^*n*^ *c*(**x** *i*) = ^*n*^ *c* *i* are coefficients ensuring the normalization of the parameters (6); both these coefficients are precomputed ahead of the shading correction. From the mean preserving condition (5) and from (7), we have

(8) 1Θ∑x∈Ωs(x)=1Θ∑x∈Ω{s(x)[1+∑i=1KΦi(β(d)(Δxi)−    mci)  nci]},

which can be configured into

(9) 1Θ∑i=1K{Φici  n[∑x∈Ωs(x)(β(d)(Δxi)−ci  m)]}=0.

The nontrivial solution (*Φ_i_* *≠* 0) of (9) is

(10) ∑x∈Ωs(x)(β(d)(Δxi)−ci  m)=0, ∀i,

which provides

(11) ci  m=∑x∈Ω s(x)β(d)(Δxi)∑x∈Ω s(x)

for each mean preserving constant *^m^c_i_* corresponding to a control point *i* with coordinates **x** *_i_*.

 Similarly, we can derive the normalization condition from (6) and(7)

(12) 1Θ∑x∈Ω|s(x)(β(d)(Δxi)−ci  m)  nci|=1, ∀i

from where

(13)     nci=1Θ∑x∈Ω|s(x)(β(d)(Δxi)−ci  m)|,

for each normalization constant ^*n*^ *c*_*i*_. Details of normalization coefficients computation in case of polynomial basis can be found in [19]; here we modified this approach for the B-spline representation. Finally, the restored image $\hat{o}\left(x\right)$ is provided using (2) by the intensity transform ${\hat{b}}^{-1}\left(x|\Phi \right)$ with the found parameter vector Φ.

Because this transformation may generally produce out-of-range intensity values, we use intermediate image representation with extended intensity range. The resultant image is finally computed using linear contrast transformation converting the intermediate image back into the original intensity range.

### 2.3. Criterion Function

In order to find parameters of the bias model describing the undesirable illumination, we need to define a criterion, with respect to which the parameters would be optimized. In the works of Likar et al. [19] and Mangin [20], the image entropy is shown to be a suitable criterion. Their idea is that the illumination is additional information added to the information included in the original signal *o*(**x**) and because we would like to remove the illumination bias, the information content of the corrected image should be lower than that of the distorted image *s*(**x**). Therefore, when looking for parameters of the reciprocal bias model eliminating the non-homogenous illumination, the Shannon's entropy *H*(.) of the resulting image $\hat{o}\left(x\right)$,

(14) $$H=-\underset{k}{\sum }\;P\left(k\right)\log\;\left(P\left(k\right)\right),$$

which is a measure of the information amount, should be minimized. Here, *P*(*k*) is the probability of intensity *k* appearing in any pixel of $\hat{o}\left(x\right)$.

Although the brightness *k* is a discrete variable in reality (represented by 8 bits, i.e., 256 values), it may be well approximated by continuous variable **κ** with a probability density *p*(**κ**). Then, in order to analytically derive the derivatives of the criterion, we may assume that the amount of information can be described by the integral version of (14) for the continuous variable **κ** as

(15) $$H=-\int_{\kappa }p\left(\kappa \right)\log\;\left(p\left(\kappa \right)\right)d\kappa .$$

**Figure 4:**
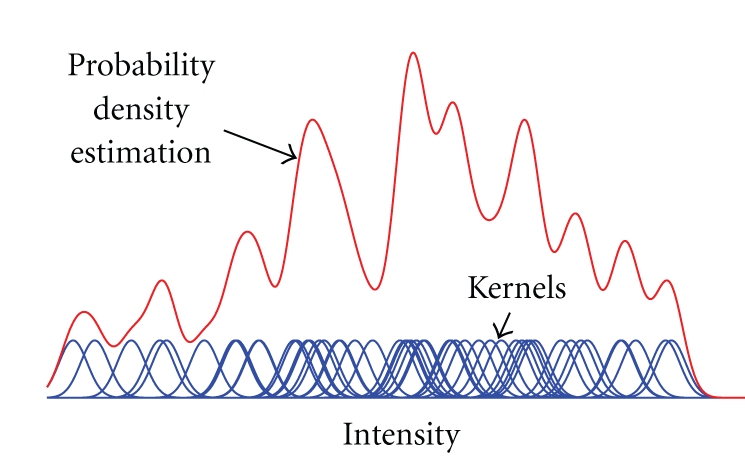
Illustration of Parzen windowing method for estimating density probability of image intensities.

However, the probability density *p*(**κ**) is not available explicitly and must be estimated from the image data transformed by the current parameters **Φ**, providing that the image has been generated by a homogeneous stochastic field. For this purpose, we used the Parzen windows (PW) technique [20], also known as kernel density estimator. In this scheme, the density is constructed by taking intensity samples $\hat{o}\left(x;\Phi \right)$ from the transformed image and super-positioning kernel functions *β* ^(3)^ centered on the elements of Ω as illustrated in the Figure 4. More formally,

(16) $$p\left(\kappa ;\Phi \right)=\frac{1}{\Theta }\underset{x\in \Omega }{\sum }\beta^{\left(3\right)}\left(\frac{\hat{o}\left(x;\Phi \;\right)}{z}-\kappa \right)$$

is the probability density estimate at intensity value **κ**, which is obtained using PW, *β* ^(3)^ is 3rd order B-spline kernel, *z* is a constant parameter defining the width of the intensity class by controlling width of the super-positioned B-spline kernel (analogue to histogram bin size), and Θ is a normalization coefficient assuring that the integral of *p*(**κ**) is equal to one. This method can be looked at like at a convolution of impulses at different intensities with a spline kernel (see Figure 4). The advantage of this method is the possibility of expressing the probability derivatives analytically and also the possibility to speed-up the computation by taking into account only a subset of the whole sample set defined in the region of interest Ω.

Thanks to our formulation of the criterion (15) based on PW, both the probability estimation and the intensity transformation are defined continuously and therefore we can analytically derive partial derivatives of the criterion with respect to components of the parameter vector **Φ**. As *H* is a compound function of the form

(17) $$H\left(\kappa ;\Phi \right)=f\left(p\left(\beta \left(\hat{o}\left(\Phi \right)\right)\right)\right),$$

its derivative with respect to an element of **Φ** can be expressed as

(18) $$\frac{\partial H\left(\kappa ;\Phi \right)}{\partial \Phi_{k}}=\frac{\partial H}{\partial p}\frac{\partial p}{\partial \beta }\frac{\partial \beta }{\partial \hat{o}}\frac{\partial \hat{o}}{\partial \Phi_{k}}.$$

From here on, the *i*th control point (a linearly decoded control point of the rectangular grid) is characterized by a vector index **k** = [*k*_1_, *k*_2_] with appropriate indices along both axes. If *n_x1_* is the number of control points along the first dimension, then * i*=*k*_1_+*n *_x1_ *k*_2_.

By applying product rule, we obtain from (15)

(19) $$\frac{\partial H\left(\kappa ;\Phi \right)}{\partial p}=-\int_{\kappa }\left(\log\;\left(p\left(\kappa ;\Phi \right)\right)+1\right)d\kappa .$$

Calculation of this integral is approximated using the rectangle rule as

(20) $$\frac{\partial H\left(\kappa ;\Phi \right)}{\partial p}\approx -\underset{k}{\sum }\left(\log\;\left(p\left(\kappa_{k};\Phi \right)\right)+1\right).$$

Further from (16),

(21) $$\frac{\partial p}{\partial \beta }=\underset{x\in \Omega }{\sum }\frac{1}{\Theta }.$$

The derivative of the B-spline kernel can be expressed using B-spline properties [27] as

(22) $$\frac{\partial \beta^{\left(3\right)}\left(\xi \right)}{\partial \xi }=\beta^{\left(2\right)}\left(\xi +\frac{1}{2}\right)-\beta^{\left(2\right)}\left(\xi -\frac{1}{2}\right).$$

The image $\hat{o}\left(x;\Phi \right)$ derived from the observed image *s*(**x**) by application of the intensity transform with parameters Φ can be expressed using (2) and (7) as

(23) o^(x;Φ)=s(x){1+∑xi∈PΦ(xi)[(β(n)(Δxi)−c(xi)  m) mc(xi)]},           where  Δxi=x−xih

and for its partial derivatives we can write (in two dimensions):

(24) ∂o^(x;Φ)∂Φk=∑xi∈P{s(x)1cin[β(3)(x1−xk1h1)−cim]    
×1cin[β(3)(x2−xk2h2)−cim]  }.

Finally, we have for the derivatives of the criterion

(25) $$\frac{\partial H\left(\kappa ;\Phi \right)}{\partial \Phi_{\text{k}}}\approx -\underset{\kappa }{\sum }\frac{\partial p\left(\kappa ;\Phi \right)}{\partial \Phi_{\text{k}}}\left(\log\;\left(p\left(\kappa ;\Phi \right)\right)+1\right),$$

where

(26) $$\begin{array}{cc} & \frac{\partial p\left(\kappa ;\Phi \right)}{\partial \Phi_{k}}\\ & =\underset{x\in \Omega }{\sum }\frac{1}{\Theta }\{{\underset{x_{i}\in P}{\sum }\frac{\partial \beta^{\left(3\right)}\left(\xi \right)}{\partial \xi }|}_{\xi =(s(x)b^{-1}(x)/z)-\kappa }\\ & \;\;\;\;\times s\left(x\right)\frac{1}{{}^{n}c_{i}}\left[\beta^{\left(3\right)}\left(\frac{x_{1}-x_{k_{1}}}{h_{1}}\right)-{}^{m}c_{i}\right]\frac{1}{{}^{n}c_{i}}\\ & \;\;\;\;\times \left[\beta^{\left(3\right)}\left(\frac{x_{2}-x_{k_{2}}}{h_{2}}\right)-{}^{m}c_{i}\right]\}. \end{array}$$

**Figure 5:**
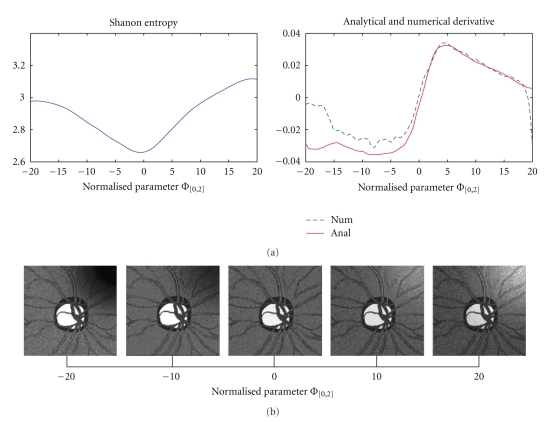
(a): Shannon entropy-based criterion and its partial derivative as functions of the parameter Φ_[0,2]_ (control point in upper-left image corner) (b): Examples of influence of Φ_[0,2]_ varying from *‒*20 to 20 with step 10, which primarily influences the upper right corner correction; notice the optimum illumination correction for the value of Φ_[0,2]_ corresponding to the zero derivative position

The grid controlling the compensation field is chosen adequately sparse as required by the smooth character of the to be compensated illumination unevenness. An example illustrating behaviour of the criterion function *H*(*κ*, Φ) as dependent on one element of the parameter vector—concretely Φ_[0,2]_ at *i* = 2, that is, for *k* = [0, 2]—top-right image corner on 3 × 3 grid covering the image—is depicted on Figure 5(a) showing a distinct minimum. Below, also the course of partial derivative of *H*(*κ*, Φ) is shown, both as derived analytically by the described approach and as calculated numerically via finite differences. There is a good agreement between both versions namely, in the important area around the; however, important advantages of analytical derivatives are faster evaluation and smooth behavior in the parameter space. On Figure 5(b), the influence of this parameter on the illumination correction is illustrated for five particular values of Φ_[0,2]_ influencing namely the correction in the upper right corner of the image; obviously, the best corrected image corresponds to the minimum of the criterion thus to the zero position of its derivative.

### 2.4. Optimization

The overall illumination correction algorithm is based on the reciprocal illumination model *b^−1^* with the parameters minimizing the entropy criterion *H*. The optimization aims at finding the optimum parameter vector

(27) $$\Phi_{\text{opt}}=\arg\underset{\Phi }{\min\;}\left\{H\left(s\left(x\right)b^{-1}\left(x\right)\right)\right\}.$$

**Figure 6:**
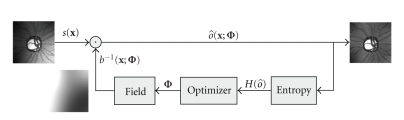
Flow diagram of iterative optimization algorithm.

The block diagram of the iterative algorithm is depicted on Figure 6.

Various types of optimization techniques were studied in order to find the optimizer best suited for the particular properties of the used optimization criterion in the problem of non-homogenous illumination correction. The tested methods were namely, downhill simplex [28] (Amoeba), Powell's direction set [28], controlled random search (CRS) [29], gradient descent (GD) [28, 30], conjugate gradient (CG) [28], and two versions of the limited memory Broyden, Fletcher, Goldfarb, Shannon (LBFGS) methods [31]. 

The comparison of results of the individual methods can be found in the next paragraph.

## 3. Experiments and Results

The proposed algorithm is supposed to be able to deal with non-homogenous illumination of retinal image data (384×384 pixel, 10 *μ*m/pixel) obtained by means of the CSLO (Heidelberg Retinal Tomograph HRT II). In order to check the efficiency of the designed method on images with a known illumination inhomogeneity, a model of the HRT II image signal was designed, using segmentation of real HRT II images into five object classes with the estimated characteristic intensity values assigned to the objects as follows.

**Figure 7:**
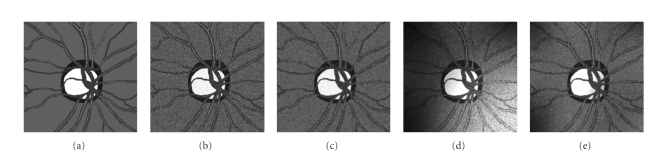
(a): Ideal model image created by manual segmentation of a real image, (b): Model image after noise addition, (c): Model image after noise addition and convolution with PSF of the imaging system. (d): Final model image corrupted by non-homogenous illumination. (e): the image after processing by the presented algorithm.

0—black background introduced by HRT II; 60—vessels; 120—vessel centers (brighter due to reflections); 90—retinal tissue; 30—optic disc; 250—optic disc cup). Further, according to the image acquisition model (1), random tissue noise *n*_tiss_ (uniformly distributed with standard deviation *σ* = 28) was added and the resulting image was convolved with the estimated point spread function of the real imaging system, approximated by 2D Gaussian kernel with standard deviation *σ* = 1. Finally, an artificial multiplicative illumination field controlled by a mesh of 3×3 nodes on the image area with random parameters was applied (see Figure 7).

A set of 40 simulated images was created via this model. Normalized parameters of the illumination field were uniformly distributed on interval [−15, 15]. Then, various optimizers were tested and evaluated with respect to achieved quality of the retrospective compensation of the illumination unevenness. The compensation quality was quantified using the fact that the known artificially introduced multiplicative illumination field and the final illumination correction field should be ideally inverse. Therefore, we can define the postcorrection illumination error *ξ*_post_ as

(28) $$\xi_{\text{post}}=\frac{1}{\Theta }\underset{x\in \Omega }{\sum }\sqrt{{\left(1-b\left(x\right){\hat{b}}^{-1}\left(x\right)\right)}^{2}},$$

where *b*(**x**) is the introduced illumination field, ${\hat{b}}^{-1}\left(x\right)$ is the found correction of the illumination, and Θ is the size of the image domain Ω. The mean precorrection illumination error defined as

(29) $$\xi_{\text{pre}}=\frac{1}{\Theta }\underset{x\in \Omega }{\sum }\sqrt{{\left(1-b\left(x\right)\right)}^{2}}$$

was set to *ξ*_pre_ = 0.2875, that is, the intensity of the ideal image was corrupted on average by approximately 30%.

**Figure 8:**
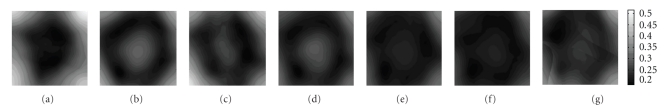
(a) Artificially introduced illumination field. Error illumination field (illumination and restoration field multiplied, ideally black) using: (b) Amoeba optimizer, (c) Powel optimizer, (d) CRS, (e) LBFGS optimizer, (f) Gradient descent optimizer (g) Conjugate gradient optimizer.

The seven optimization algorithms mentioned in the previous section were tested and the average results obtained when using the simulated image set are summarized in Table 1. Here, the achieved after-correction total mean errors are presented, together with the computing times per image, numbers of iterations, and the achieved percentage suppression of the illumination unevenness. Examples of error fields resulting from these optimizers can be seen on Figure 8. The Amoeba, Powell and CRS methods need to evaluate the criterion value only; the derivatives of the criterion need not be computed. On the other hand, these three methods converge slowly and the total computational demands are higher compared to GD, CG, and LBFGS. Moreover, the worst problem of Amoeba, CRS, and especially of Powell algorithm was the unreliable convergence. The Powell optimizer was usually unable to find the optimum parameters of the correction illumination field (see Table 1). The false convergence to a side extreme is less frequent in case of smaller distortions introduced but even in this case the gradient-based algorithms LBFGS and GD outperform the nongradient algorithms. This can be explained by smoother description of the shape of the multidimensional cost function when using the analytical derivatives. Surprisingly, the simplest gradient descent optimizer with variable step proved to be the best with respect to the correction precision, suppressing the illumination errors by about 67 per cent on average (from *ξ*_pre_ = 0.2875 to *ξ*_post_ = 0.0926). The LBFGS optimizer was the fastest but the average achieved suppression of the simulated illumination error was only 51 per cent.

In frame of experiments, we tested also the influence of different number of image samples used for probability approximation. As can be seen in Table 2, even as little as 15% (about 20 000) of the available samples are statistically powerful enough to provide good approximation of probability density of image intensities. This results in faster computation of the criterion. 

**Figure 9:**
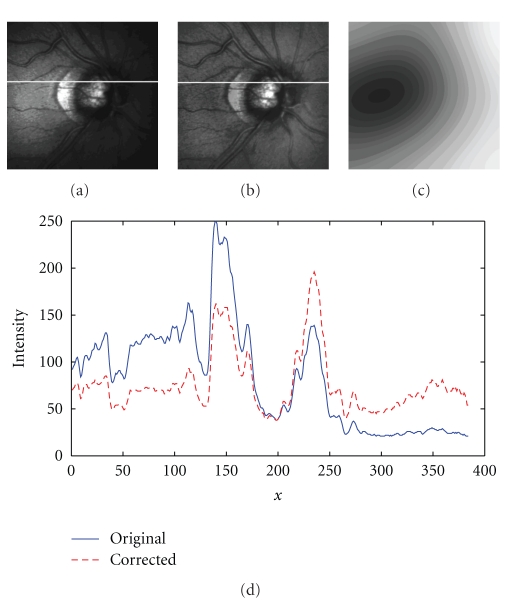
(a) Retinal image highly corrupted by non-homogenous illumination, (b) Image after multiplicative correction by recovered bias field, (c) Normalized bias field controlled by 3×3 parameters automatically obtained using proposed algorithm, (d) Intensity profiles along the indicated row.

The next set of experiments concerned real retinal images with clearly visible illumination inhomogeneities that however were not known. The algorithm was tested on the set of 336 real (clinically obtained) retinal images. Obviously, the quality evaluation of non-homogenous illumination compensation could only be performed subjectively, due to lack of the golden standard (i.e., ideal illumination in each case). In majority of cases (about 95%), a substantial improvement was visible; in the remaining cases, the image remained practically unchanged, that is, no image was further distorted by the correction. On Figure 9, a result of the designed algorithm as applied to a real retinal HRT II image is illustrated; the intensity profiles clearly show the successful bias correction. 

## 4. Conclusions

A method for efficient illumination correction was proposed, implemented, and verified: quantitatively on simulated images with known deterioration and qualitatively on an extensive set of real retinal images lacking this knowledge. It is based on estimating a B-spline polynomial shading model, the inversion of which provides correction of the input image, optimal in sense of the used information-based criterion—Shannon's entropy. Previously published methods using a similar principle were modified namely, as to the type of the used correction function concerns, and as a novelty, the computation of the criterion is based on probability distribution estimates by Parzen windowing method, which enables us to derive analytical expressions for derivatives of the criterion. This consequently substantially speeds up the computations. Along with the efficient B-spline distortion model, it results in the possibility of efficient usage of gradient-based optimizers. We compared the efficiency and precision of three non-gradient and four gradient-based optimizers and found the classical gradient descent optimizer with variable step as the best for the purpose of illumination correction, formulated in the suggested way.

 The quantitative tests were done on an image set artificially created with respect to characteristics of the imaging device (the method is primarily aimed at improving quality of retinal images taken by means of the HRT II CSLO); these tests have shown that the designed algorithm is capable of removing up to about 70% of artificially introduced illumination variability. Finally, the method was successfully qualitatively tested on a set containing 336 real CSLO clinically obtained retinal images.

According to results of some further tests, involving subsequent registration of multiple images, preprocessing of the retinal images by the proposed algorithm had a clearly positive influence on reliability of the registration of the images using the registration method developed by our group [1], when compared to registration results concerning the unprocessed images. A similar positive effect can be expected for even higher types of image analysis following the presented correction method.

## Figures and Tables

**Table 1:** Study of suitability of different optimizers for the task of optimization of Shannon's entropy. The best values are highlighted.

	Nongradient-based methods			Gradient-based methods			
	Amoeba	Powell	CRS	L-BFGS	L-BFGS-B	**GD**	CG
*ξ*_*post*_	0.16	0.23	0.14	0.13	0.14	**0.09**	0.18
*t* [s]	68.6	95.6	514	14.7	**15.5**	45.9	410
N	163	226	1201	26.4	**21.0**	57.4	615
*ρ* [%]	44.5	18.6	52.9	55.9	51.3	**67.7**	36.9

*ξ*_pre_ = 0.28.

**Table 2:** Influence of different number of samples used for entropy evaluation on the algorithm precision and speed.

GD, 50 bins			
coverage	0.15	0.5	1
*ξ*_post_	**0.09**	**0.09**	**0.09**
*t* [s]	45.8	46.1	212
*N*	57.4	57.5	67.6
